# Renal outcomes with sodium-glucose cotransporters 2 inhibitors

**DOI:** 10.3389/fendo.2022.1063341

**Published:** 2022-12-01

**Authors:** Xiaoya Sun, Guohong Wang

**Affiliations:** Department of Geriatrics, Beijing Tongren Hospital, Capital Medical University, Beijing, China

**Keywords:** SGLT2 inhibitors, kidney, eGFR, albuminuria, type 2 diabetes

## Abstract

Diabetic nephropathy (DN) is one of the most serious complications of diabetes. Therefore, delaying and preventing the progression of DN becomes an important goal in the clinical treatment of type 2 diabetes mellitus. Recent studies confirm that sodium-glucose cotransporters 2 inhibitors (SGLT2is) have been regarded as effective glucose-lowering drugs with renal protective effect. In this review, we summarize in detail the present knowledge of the effects of SGLT2is on renal outcomes by analyzing the experimental data in preclinical study, the effects of SGLT2is on estimated glomerular flitration rates (eGFRs) and urinary albumin-creatinine ratios (UACRs) from clinical trials and observational studies, and renal events (such as renal death or renal failure requiring renal replacement therapy) in some large prospective cardiovaslucar outcomes trials. The underlying mechanisms for renoprotective activity of SGLT2is have been demondtrated in multiple diabetic and nondiabetic animal models including kidney-specific effects and secondary kidney effects related to amelioration in blood glucose and blood pressure. In conclusion, these promising results show that SGLT2is act beneficially in terms of the kidney for diabetic patients.

## Introduction

Type 2 diabetes (T2D) is a rapidly increasing global health concern throughout the world. According to the 2021 International Diabetes Federation (IDF) statistics, the number of diabetic patients among the age group of 20-79 years worldwide is 537 million. This number is predicted to rise to 643 million by 2030 and 783 million by 2045 (1). Thus, diabetes imposes a substantial economic burden on the whole society.The primary characteristic of T2D involves chronic hyperglycemia, which can correspondingly cause the development of chronic complications, such as cardiovascular and renal events. Over the past 30 years, although the cardiovascular complications in T2D have progressively declined, the incidence of end-stage renal disease (ESRD) has not considerably changed (2). Therefore, new therapeutic requirements for the current hypoglycemic strategy should be considered, which can not only lower blood glucose levels, but also prevent cardiovascular and kidney damage or delay their progression. Based on these requests, some large-scale clinical trials of a new class of hypoglycemic drugs have recently provided ideal information regarding these endpoints, with reduced major adverse cardiovascular and renal events being reported (3, 4). These findings have resulted in renal protection related to diabetic treatment receiving increasing degrees of attention. Although some antihyperglycaemic agents (AHAs), such as incretin-based therapies (e.g. glucagon-like peptide-1 receptor agonists, dipeptidyl peptidase-4 inhibitors), have been proven to improve renal function in diabetic nephropathy (DN) patients (5–8), their use in these patients is restricted owing to reduced efficacy and/or side effects. Thus, a novel therapeutic option aimed at improving clinical outcomes in this high-risk population are therefore highly desirable.

Sodium-glucose cotransporters 2 (SGLT2) inhibitors are a new class of oral anti-diabetic drugs that have played an increasingly important role in the management of type 2 diabetes in recent years (9–11). Under normal physiological conditions, ~90% of the glucose filtered by the glomerulus is reabsorbed into the body by SGLT2 on the renal proximal tubule. A SGLT2 inhibitor (SGLT2i) acts directly on the kidney by inhibiting SGLT2 function thereby blocking the reabsorption of glucose and expelling excessive glucose in the urine, which eventually reduces hyperglycemia (12). The anti-hyperglycemic effect of SGLT2i, which is different from many other antidiabetic agents, is a completely insulin-independent mechanism of action, which allows for the drugs to be used both in the early and late stages of diabetes (13). Furthermore, SGLT2i improves glucose control without inducing hypoglycemia, thus making it a favorable clinical drug option (9). Of particular note is the recent large clinical trials focusing on some SGLT2is in T2D patients with a high risk of cardiovascular events, which have demonstrated a reduction in cardiovascular and renal adverse outcomes (3, 4). In the EMPA-REG OUTCOME trial, T2D patients at high cardiovascular risk treated with empagliflozin had a slower progression of renal diseases and lower incidences of clinical-related renal events (14). Similar results have been reported with canagliflozin treatment in the CANVAS program (3). Hence, these data suggest that SGLT2i may be an important therapeutic option for patients with diabetic kidney diseases, although the related mechanisms of protection still remain unclear. However, due to the unique renal mechanism of action, SGLT2i have been contraindicated for patients with an estimated glomerular filtration rate (eGFR) below 45 ml per minute per 1.73 m^2^ of body-surface area (9).

**Figure 1:**
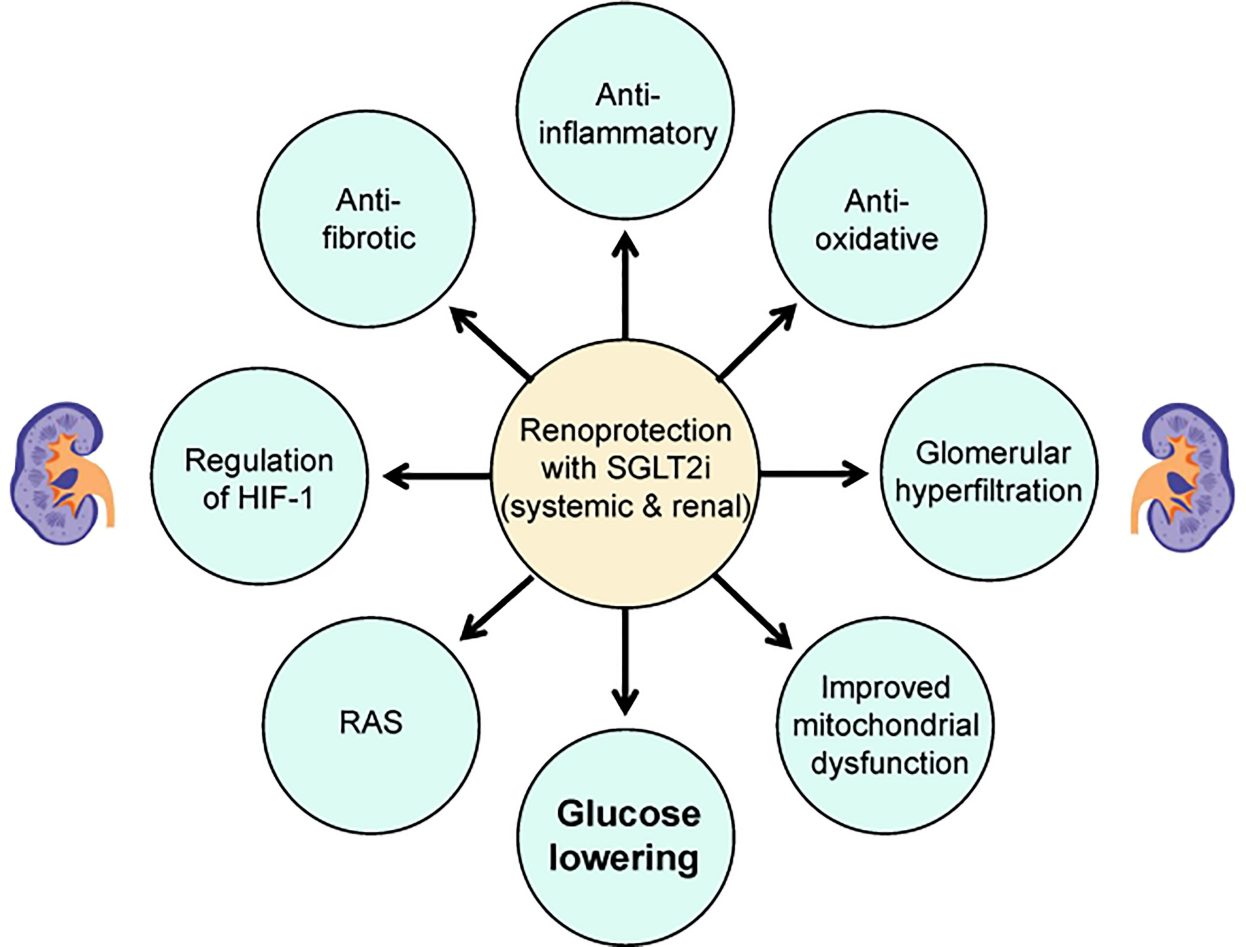
Potential mechanisms of renoprotective effects of SGLT2i in patients with type 2 diabetes. HIF-1, hypoxia-inducible factor 1; SGLT2i, sodium-glucose cotransporter-2 inhibitors; RAS, renin-angiotensin system.

In addition to the attenuated antihyperglycemic effects, the enhanced urinary glucose excretion also contributes to body weight loss and blood pressure reduction (15, 16). Persistent glomerular hyperfiltration is considered to be involved in the occurrence and development of diabetic nephropathy, and SGLT2i have been found to have a biphasic effect on glomerular hyperfiltration, including an initial decrease in eGFR followed by a stabilized preservation (17). Furthermore, SGLT2i effectively reduce albuminuria despite the different levels of eGFR, which is independent of coincident changes in hemoglobin A1c (HbA1c), blood pressure and body weight (18–20). The reduced glucose absorption through the renal proximal tubule may attenuate the hyperglycemia-related tubulointerstitial injury and correspondingly assist with the protection of the diabetic kidney (21, 22). Experimental studies in various animal models have shown that the potential renal protective properties of SGLT2i may include a reduction in inflammation, oxidative stress, apoptosis and fibrosis (**Figure 1** and **Table 1**).

**Table 1:** Mechanisms of renoprotective effects of sodium-glucose cotransporters 2 (SGLT2) inhibitors in studies of multiple animal models.

Mechanisms	References
Reduction of inflammation	(21, 23–28)
Reduction of oxidative stress	(21, 23–25, 28–34)
Reduction of apoptosis	(21, 23, 25, 29, 35)
Inhibition of glomerulosclerosis and interstitial fibrosis	(23–25, 27, 36–40)
Alleviation of glomerular hyperfiltration	(41)
Suppression of renal lipid accumulation	(24, 25)
Attenuation of mesangial cell widening/proliferation	(27, 42)
Suppression of renin-angiotensin system (RAS)	(31, 42)
Regulation of hypoxia-inducible factor 1 (HIF-1)	(43–45)
Improvement in mitochondrial dynamic and autophagy	(46)
Reduction of renal artery stiffness	(47)
Suppression of tricarboxylic acid cycle intermediate	(34)

The SGLT2i are proposed as promising candidates for treating hyperglycemia, and the prevention of the onset and progression of renal adverse events in diabetes despite the kidney-protective mechanism deserves more investigation. Therefore, this review summarizes the evidence of renal outcomes with SGLT2i from basic animal studies, human clinical trials and recent large prospective randomized controlled studies. Moreover, the current review focuses only on the six commercially available SGLT2i (dapagliflozin, empagliflozin, canagliflozin, ipragliflozin, tofogliflozin and loseogliflozin), and does not include SGLT2i that are still under development.

## Quality assessment and data extraction

All studies evaluating the association between SGLT2 inhibitors and kidneys were selected.

The review was performed in accordance with the Preferred Reporting Items for Systematic Reviews and Meta-Analyses (PRISMA) statement. Studies were initially screened on the basis of their title or abstract. Studies that seemed to meet the qualification criteria were selected for full text review. Studies that did not include data on SGLT2 inhibitors and kidneys were excluded.

## Dapagliflozin

### Mechanistic studies of animal experiments

Dapagliflozin exerts renoprotective effects in various animal models, including progressive diabetic nephropathy rats (23, 35, 41), high fat diet (HFD)-induced obese (24) or prediabetic rats (25), rats with either type 1 diabetes (T1D) (36) or T2D (42), db/db mice with uninephrectomy (37), gentamicin-induced nephrotoxic rats (29) and ischemia-reperfusion mice (43).

Some mechanisms may explain this kidney-protective effect of dapagliflozin, among which are reductions of inflammation and oxidative stress (23–25, 29, 35, 37, 42), reduction of apoptosis (23, 25, 29, 35), alleviation of glomerular hyperfiltration (41), attenuation of fibrosis by regulating STAT1/TGFβ1 signaling (36), suppression of the renin-angiotensin system (RAS) (42), and induction of hypoxia-inducible factor 1 (HIF1) (43) (**Figure 1** and **Table 1**).

However, inconsistent with these studies, a study in progressive chronic kidney disease (CKD) rats with 5/6 (subtotally) nephrectomy did not show any renoprotective effects with dapagliflozin treatment. Additionally, neither the decreased eGFR nor the extent of glomerulosclerosis and tubulointerstitial fibrosis was improved, thus suggesting that dapagliflozin does not attenuate disease progression in the classical kidney model of non-diabetic CKD (48).

### Effects on renal markers in patients with T2D

Numerous studies have verified that the reduction in albuminuria with dapagliflozin treatment appears to be independent of changes in HbA1c, eGFR or systolic blood pressure (SBP). In two multicenter randomized controlled trial (RCT) studies, patients with T2DM and hypertension were assigned to either dapagliflozin 10 mg/day or placebo added to an ongoing RAS blockade therapy (20). After 12 weeks, eGFR declined with dapagliflozin treatment, which was completely reversible at 1 week after the discontinuation of treatment. Dapagliflozin significantly reduced albuminuria that was independent of changes in either glucose or blood pressure control (20) (**Table 2**). Furthermore, a longer-term (104-week) study in patients with T2D and stage 3 CKD found that dapagliflozin treatment was associated with an improvement in the urinary albumin:creatinine ratio (UACR) (-57.2% and -43.8% for those with dapagliflozin at 10 mg and 5 mg, respectively, at 104 weeks); however, these changes in UACR did not correlate with those changes in glycemic control (19). An initial decrease was observed in eGFR with the first 4 weeks of dapagliflozin therapy and no further decline occurred over the 104 weeks, whereas a gradual decline was observed in the placebo-treated group (19). In a 102-week RCT, compared with the placebo group, UACR was reduced and eGFR remained unaffected with dapagliflozin treatment, despite decreases in HbA1C in T2D patients with stage 3b-4 (49) (**Table 2**). Thus, these studies suggest that dapagliflozin treatment has potential renal benefits for diabetic patients at high renal risks (19, 49).

**Table 2:** Effects of Sodium-glucose cotransporters 2 (SGLT2) inhibitors on urinary albumin creatinine ratio (UACR) in randomized controlled trials (RCTs) in patients with type 2 diabetes and chronic kidney disease (CKD) of different levels.

SGLT2inhibitors	Reference	Type of kidney disease patient	Duration(weeks)	Dose(mg/day)	Comparator	Patients (n)	Baseline UACR(mg/g ± SD or IQR)	Change UACR(% vs. baseline or placebo)	*P*
Dapagliflozin	20	UACR ≥ 30mg/g	12	10mg	Placebo	167 vs 189	Placebo: 78.0 (44.0 to 267.0) Dapa: 75.0 (44.0 to 267.0)	-33.2 vs Placebo (95%CI: -45.4 to -18.2)	NA
Dapagliflozin	34	UACR ≥ 30mg/g	24	5mg	Placebo	33 vs 29	Placebo: 52.0 (17.0 to 180.0)		NA
				10mg		41 vs 29	5mg: 51.0 (18.0 to 539.0)	5mg vs Placebo: 4.9 (95%CI:-68.5 to -20)	
							10mg: 40.0 (9.0 to 285.0)	10mg vs Placebo: -34.6 (95%CI:-57.6 to -0.9)	
Canagliflozin	65	UACR: 30-2000mg/g eGFR: 45-89ml/min/1.73m^2^	52	100mg	other ADs	20 vs 20	other: 159 (58 to 1156) Cana: 139 (67 to 1506)	-83 vs 27	0.004
Canagliflozin	66	eGFR: 30-50mg/g	52	100mg 300mg	Placebo	90 vs 90 89 vs 90	Placebo: 25.9 100mg: 22.1 300mg: 37.3	-0.9 vs 2.2 -6.9 vs 2.2	NA
Canagliflozin	67	UACR ≥ 30mg/g	104	100mg	Glimepiride	76 vs 76	Glim: 60.1 (41.29 to 124.91)		
				300mg		78 vs 76	100mg: 56.5 (40.17 to 115.49)	100mg vs Glim: -31.7	0.01
							300mg: 75.2 (43.35 to 173.11)	300mg vs Glim: -49.3	< 0.001

NA, not available; eGFR, estimated glomerular filtration rate; ADs, antidiabetic drugs.

In a real-world study with 17,258 T2D patients who received dapagliflozin or other glucose-lowering agents, the albumin excretion rate was reduced in the dapagliflozin group and remained unchanged in the other groups. Although dapagliflozin resulted in a mild decline in eGFR, there was no evidence of worsening renal function (50). Another study was conducted in patients with T2D and residual albuminuria on stable doses of angiotensin-converting enzyme inhibitors or angiotensin receptor blockers who were exposed to 10 mg/d dapagliflozin or placebo for 2 consecutive treatment periods of 6 weeks, with a 6-week wash-out period occuring in between the treatment (51) (**Table 3**). Dapagliflozin markedly reduced 24 h urinary albumin excretion (UAE), which was reversible after the discontinuation of treatments, and the albuminuria-lowering effect was reproducible upon re-exposure to dapagliflozin, thus indicating that dapagliflozin treatment is associated with a reduction in albuminuria. Furthermore, there was a significant correlation between improvements in proteinuria and in morning home SBP of add-on dapagliflozin therapy in Japanese patients with T2D and diabetic nephropathy (52).

**Table 3:** Effects of Sodium-glucose cotransporters 2 (SGLT2) inhibitors on estimated glomerular filtration rate (eGFR) in randomized controlled trials (RCTs) in patients with type 2 diabetes and chronic kidney disease (CKD) of different levels.

SGLT2inhibitors	Reference	Type of kidney disease patient	Duration(weeks)	Dose(mg/day)	Comparator	Patients (n)	Baseline eGFR(ml/min/1.73m^2^)	Change eGFR(ml/min/1.73m2 vs.baseline or placebo)	*P*
Dapagliflozin	20	UACR ≥ 30mg/g	12	10mg	Placebo	167 vs 189	Placebo: 85.8 ± 21 Dapa: 82.1 ± 19.7	-4.8 vs -2.8	NA
Dapagliflozin	34	eGFR: 12-45ml/min/1.73m^2^	102	5mg 10mg	Placebo	58 vs 69 93 vs 69	Placebo: 38.4 5mg: 37.6 10mg: 38.0	no difference	NA
Dapagliflozin	36	UACR: 100-3500mg/g eGFR ≥ 45ml/min/1.73m^2^	6	10mg	Placebo	33	72	-4.8 vs 0.9	< 0.001
Canagliflozin	65	UACR: 30-2000mg/g eGFR: 45-89ml/min/1.73m^2^	52	100mg	other ADs	20 vs 20	Other: 55.4 ± 12.3 Cana: 57.1 ± 16.2	0.7 vs -3.4	0.024
Canagliflozin	66	eGFR: 30-50ml/min/1.73m^2^	52	100mg 300mg	Placebo	90 vs 90 89 vs 90	Placebo: 40.1 (6.8) 100mg: 39.7 (6.9) 300mg: 38.5 (6.9)	-0.5 vs Placebo -2.5 vs Placebo	NA
Canagliflozin	67	UACR ≥ 30mg/g	104	100mg 300mg	Glimepiride	76 vs 76 78 vs76	Glim: 86.5 (18.5) 100mg: 91.0 (23.9) 300mg: 91.1 (23.9)	0.1 vs -2.7 0.1 vs -2.7	< 0.001 < 0.001

UACR, urinary albumin creatinine ratio; NA, not available; ADs, antidiabetic drugs.

In a *post-hoc* analysis of an RCT, dapagliflozin significantly reduced albuminuria in patients with T2D, and changes in albuminuria were correlated with the reduction in eGFR, as well as with the decrease in urinary tubular marker-kidney injury molecule-1 (KIM-1) (53). Thus, the mechanism by which dapagliflozin reduces albuminuria may be the result of reduced intraglomerular pressure and improved tubular cell integrity. Another study in 40 patients with hypertension and T2D found that there was a significant correlation between the renal function-related miRNAs (such as miR130b-3p and miR21-5p) and dapagliflozin treatment (54). These findings implied that a possible mechanism of nephroprotection with dapagliflozin involved epigenetic regulation, which warrants further investigation in a larger sample size.

### Renal outcomes in clinical studies

In the DERIVE study (NCT02413398), patients with T2D and CKD 3a (eGFR 45-59 mL/min/1.73m^2^) were randomized to dapagliflozin (10 mg once daily) or placebo groups for 24 weeks (55). Compared with the placebo group, dapagliflozin significantly improved HbA1c over 24 weeks of treatment. Moreover, a transient drop in eGFR from baseline was observed with dapagliflozin treatment, which was reversible after the discontinuation of treatment. The change in eGFR was similar to findings in patients with normal or mild renal impairment, for whom dapagliflozin treatment is associated with reversible decreases in eGFR and long-term eGFR stabilization (56). In addition, although there were no significant difference in UACR changes at week 24 between the dapagliflozin and placebo groups, a decrease in UACR was observed in patients with baseline UACR ≥ 30 mg/g (**Table 4**). The limitation of this study included the relatively short duration of the study and the long-term effects of dapagliflozin are needed. In summary, this study supports a positive benefit of dapagliflozin in T2D patients with stage 3a CKD.

**Table 4:** Renal outcomes in type 2 diabetes patients treated with Sodium-glucose cotransporters 2 (SGLT2) inhibitors in five major cardiovascular outcome trials.

Renal endpoints	DERIVE (55)(dapagliflozin)	DECLARE (57)(dapagliflozin)	EMPA-REG Outcome (14) (empagliflozin)	CANVAS (58, 59)(canagliflozin)	CREDENCE (60, 61)(canagliflozin)
Baseline eGFR (ml/min/1.73m^2^)	53.3 ± 8.7	subgroup 98.3 77 51.4	subgroup 48.6 ± 7.8 82.7 ± 16.6	76.5 ± 20.5	56.2
Change in eGFR (ml/min/1.73m^2^, placebo-subtracted)	-2.49 (-4.96 to -0.02) NA	*P* < 0.0001 *P* < 0.0001 *P* = 0.053	10mg: 0.48 ± 0.04 25mg: 0.55 ± 0.04 placebo: 0.04 ± 0.04 *P* < 0.0001	NA	1.52 (1.11 to 1.93) *P* < 0.001
Baseline UACR (median mg/g)	23.5 (2.7 to 5852.0)	NA	subgroup < 30 NA 30-300 NA > 300 NA	12.3 (6.65 to 42.1)	927 (463 to 1833)
UACR (mg/g or %, placebo-subtracted)	8% (-14.4% to 36.3%) *P* = 0.513	NA	47.8‰ vs 76‰	NA	31% (26% to 35%) *P* < 0.01
Progression to macroalbuminuria (HR)	NA	NA	0.62 (0.54 to 0.72) *P* < 0.0001	0.73 (0.67 to 0.79)	NA
Doubling of serum creatinine (HR)	NA	NA	0.56 (0.39 to 0.79) *P* < 0.0001	NA	0.6 (0.48 to 0.76) *P* = 0.16
Sustained 40% reduction in eGFR (HR)	NA	0.54 (0.43 to 0.67) *P* < 0.0001	NA	0.53 (0.33 to 0.84) *P* > 0.05	NA
Continuous renal replacement therapy (RRT)	NA	NA	0.45 (0.21 to 0.97) *P* = 0.04	NA	NA
ESRD or renal death (HR)	NA	0.41 (0.2 to 0.82) *P* = 0.012	NA	NA	NA
Composite renal outcomes (HR or %)	0.6% vs 1.2%	0.53 (0.43 to 0.66) *P* < 0.0001	0.54 (0.4 to 0.75) *P* < 0.001	0.6 (0.47 to 0.77)	0.7 (0.59 to 0.82) *P* = 0.11
Definition of composite renal outcomes (variable across trials)	Renal failure^a^	sustained 40% reduction in eGFR,	doubling of SCr accompanied,	sustained 40% reduction in eGFR,	chronic dialysis > 30 days,
		kidney transplantation,	by eGFR ≤ 45 ml/min/1.73m^2^,	RRT,	kidney transplanatation, eGFR< 15ml/min/1.73m^2^ > 30 days,
		sustained eGFR< 15ml/min/1.73m^2^,	RRT,	death from renal causes	doubling of SCr,
		death from renal causes	death from renal causes		death from renal causes

In all trials, the results of glucose-lowering agents were compared with placebo; data in parentheses are 95% confidence interval or percentage. eGFR, estimated glomerular filtration rate; NA, not available; UACR, urinary albumin-creatinine ratio; HR, hazard ratio; RRT, renal replacement therapy; ESRD, estimated glomerular filtration rate; SCr, serum creatinine.

^a^ Not precisely defined in the original report.

In the Dapagliflozin Effect on Cardiovascular Events (DECLARE, NCT01730534)-TIMI 58 trial, 17,160 T2D patients both with and without established atherosclerotic cardiovascular disease (CVD) and creatinine clearance of at least 60 mL/min were randomly assigned to 10 mg dapagliflozin or placebo once daily (57). After a median follow-up of 4.2 years, dapagliflozin decreased the incidence of the primary composite outcomes (cardiovascular death or hospital admission for heart failure) but did not reduce the incidence of the other outcomes (major adverse cardiovascular events). A decrease in eGFR of ≥ 40% to < 60 mL/min per 1.73 m², a secondary composite outcome, was significantly lower in the dapagliflozin group than in the placebo group. As equalized by the mean change at 2 years, 3 years and 4 years, the mean eGFR decrease was less with dapagliflozin than with the placebo. Despite the small numbers of events, ESRD or renal death occurred less frequently in the dapagliflozin group (11 of 8,582 patients) than in the placebo group (27 of 8,578 patients). Unfortunately, due to the long interval of creatinine concentrations and UACR detection, subtle changes in renal function may have been missed. Therefore, these data suggest that dapagliflozin seems to be beneficial for preventing and reducing the progression of kidney disease in T2D patients, irrespective of the presence of atherosclerotic CVD or baseline renal function (**Table 4**).

## Empagliflozin

### Mechanistic studies of basic experiments

Empagliflozin has demonstrated renoprotective effects in both diabetic and non-diabetic animal models. In T1D mice, empagliflozin was found to attenuate diabetes-induced oxidative stress, apoptosis and inflammation, thus leading to the renoprotective actions of empagliflozin (26, 46). In addition, empagliflozin ameliorated kidney injury in db/db female mice by reducing systemic and renal arteriosclerosis, as well as by suppressing the expression of fibrotic mediators (47). In spontaneously diabetic Ins2+/Akita mice, the renal protective mechanism of empagliflozin may be attributed to the regulation of adenosine pathways, which play a pivotal role in glomerular hemodynamic effects and tubuloglomerular feedback (62). In nondiabetic salt-sensitive hypertensive rats, the protective effect of empagliflozin on blood pressure was associated with increased expression of renal HIF-1α and the amelioration of inflammation in the kidneys (44). However, empagliflozin alone did not prevent hypertension in an Ang II-induced kidney damage model, but played an additional role in preventing kidney damage by decreasing reactive oxygen species generation, increasing eGFR and reducing urinary protein excretion (30) (**Figure 1** and **Table 1**).

### Effects on renal markers in patients with T2D

In a retrospective observational study, 110 Japanese T2D patients who received empagliflozin (10 mg daily) were divided into two groups: a non-elderly subject group (age ≤ 65 years) and an elderly subject group (age ≥ 65 years). At 6 months, eGFR, urinary protein excretion and blood pressure were significantly reduced only in non-elderly patients, and both the ΔeGFR and ΔUP were considerably associated with changes in systolic blood pressure, thus suggesting that the renoprotective effect of empagliflozin was correlated with blood pressure reduction, whereas this effect was limited in elderly patients because of the decreased arterial elasticity (63). Regarding the efficacy of empagliflozin in kidney transplant recipients with diabetes, studies have shown that eGFR remained stable after 6-12 months of follow-up, thus indicating that renal transplant was well tolerated in recipients with diabetes mellitus (DM) (64, 65).

In patients with T2D, the kidney-protective mechanism of empagliflozin may be to provide more renal fuel energetics by decreasing hyperfiltration and improving renal oxygenation (14, 66). Moreover, an RCT study using blood oxygenation level-dependent magnetic resonance imaging could help to explore the effects of empagliflozin (10 mg/day) on renal tissue oxygenation (67). However, this study was limited to healthy volunteers and more investigations are needed to explore the effect of empagliflozin on renal oxygenation in diabetic individuals.

### Renal outcomes in clinical studies

In the EMPA-REG OUTCOME (Empagliflozin Cardiovascular Outcome Event Trial in Type 2 Diabetes Mellitus Patients) trial, T2D patients with CVD and eGFR ≥ 30 mL/min/1.73m^2^ were assigned to empagliflozin 10 mg, 25 mg or placebo once daily. In addition to the cardiovascular benefits, empagliflozin also exerted a significant decline in important renal endpoints compared with the placebo. Empagliflozin significantly reduced the risks of incident/worsening nephropathy, including the progression to macroalbuminuria, the doubling of serum creatinine, the initiation of renal replacement therapy or renal death compared with the placebo (14, 68). The effects of empagliflozin on these outcomes were consistent across the categories of eGFR (< 45, 45 to < 60, 60 to < 90 and ≥ 90 mL/min/1.73m^2^) and UACR (> 300, 30 to ≤ 300 and < 30 mg/g) at baseline and across the two examined doses (69). In addition, there was no heterogeneity in the effect of empagliflozin on the incidence/progression of nephropathy (*P* ≥ 0.05 for the interactions) across the subgroups defined by age (< 65, 65 to <75 and ≥ 75 years) (70).

In terms of renal function, patients in the empagliflozin group had an initial decrease in eGFR (within 4 weeks), which remained stable within 188 weeks, and the eGFR was fully reversible after the discontinuation of empagliflozin treatment. However, placebo-treated recipients had a gradual, irreversible decrease in eGFR (14). Moreover, UACR was lower in empagliflozin-treated participants than in the placebo group, with effects most pronounced in those individuals with baseline microalbuminuria or macroalbuminuria (**Table 4**). Furthermore, given the potential differences in disease etiology between Asian patients and other populations, subgroup analyses of EMPA-REG OUTCOME showed that the effects of empagliflozin on the kidneys in Asian patients were consistent with those of the overall trial population findings (71).

In exception of the favorable results in the EMPA-REG OUTCOME trial, future studies in CKD patients both with and without diabetes are underway to provide important insights into the renoprotective effects of empagliflozin (such as the EMPRESS trial and EMPEROR trial). Until now, the EMPEROR-Reduced study has found that the effect of empagliflozin on the decrease in eGFR and on the risk of the composite renal outcome was consistent in patients with or without CKD, regardless of the degree of kidney impairment at baseline, which reinforces the beneficial effects of empagliflozin on delaying the progressive development of renal function decline (72, 73).

## Canagliflozin

### Mechanistic studies of animal experiments

The renal protective effects of canagliflozin have been shown in nicotinamide/streptozotocin/sucrose-induced diabetic mice (27), in New Zealand obese mice fed a HFD (31), in T2D rats with acute kidney injury after myocardial infarction (MI) (32), in cisplatin-induced nephrotoxic mice (28) and in adenine-induced renal failure mice (74). It has also been shown that canagliflozin reduces inflammation and fibrotic changes in the kidney by inducing AMP-activated kinase (AMPK) activation in T2D mice (27). Furthermore, the downregulated expression of renal angiotensinogen and the reduced oxidative stress by canagliflozin were found to mitigate high blood pressure and renal tubular fibrosis in mice with T2D (31). In a kidney injury model after MI in diabetic rats, canagliflozin demonstrated renal protection *via* the β-hydroxybutyrate mediated reduction of nicotinamide adenine dinucleotide phosphate oxidase proteins (NOXs) and oxidative stress (32). In nephrotoxic mice, canagliflozin ameliorated cisplatin-induced inflammation and improved renal histopathological damage, thus leading to a protective effect of SGLT2i (28) (**Figure 1** and **Table 1**). In addition to SGLT2 inhibition, canagliflozin exerts modest SGLT1 inhibitory activity. Moreover, the analysis of cecal microbiota demonstrated considerable changes in microbiota composition and inhibition of the accumulation of plasma uremic toxins by canagliflozin which may offer a novel potential treatment in CKD disease (74).

### Effects on renal markers in patients with T2D

A prospective, open-label study evaluated the effects of canagliflozin on renal function in Japanese T2D patients with CKD (75). After 52 weeks of treatment, the mean changes in UACR between the canagliflozin (100 mg/day) group and placebo group were -83 mg/gCr and 27 mg/gCr, respectively (*P* = 0.004; **Table 2**). However, there were more reductions in eGFR from baseline with canagliflozin at week 4, which gradually trended back to baseline over 26 weeks of treatment (**Table 3**). Furthermore, a significant reduction in tubulointerstitial markers was observed in the canagliflozin group, but not in the control group. Thus, this study suggests that canagliflozin treatment in T2D with CKD is associated with a reduction in albuminuria and tubulointerstitial biomarkers (75).

These results were confirmed by a RCT with canagliflozin (100 mg or 300 mg), which identified 269 patients with T2D and with a subset of stage 3 CKD (defined as eGFR ≥ 30 and < 50 mL/min/1.73m^2^) (76). eGFR had a small initial decrease in both canagliflozin groups, which trended back toward baseline compared with the placebo group (**Table 3**). Moreover, the mean UACR was reduced with canagliflozin 100 mg and 300 mg at week 52, whereas an increase was observed with the placebo group. The proportion of patients with progression in albuminuria declined with canagliflozin 100 and 300 mg, whereas the proportion of patients with regression in albuminuria was greater than those taking placebo (76) (**Table 2**).

In a larger and longer-term (2-year) clinical trial, 1,450 T2D patients receiving metformin were assigned to canagliflozin 100 mg or 300 mg and glimepiride 6-8 mg (77). The eGFR decline at 2 years was significantly lower in both canagliflozin groups compared with the glimepiride group (*P* ≤ 0.01; **Table 3**). The UACR remained unchanged in the canagliflozin 100 mg group and was reduced in the first-year treatment of canagliflozin 300 mg, which subsequently returned to baseline at 2 years, whereas the UACR progressively increased in the glimepiride group (**Table 2**). The albuminuria-lowering effect of canagliflozin was not influenced by HbA1c reductions, thus suggesting that the renoprotective effects may be independent of changes in glycemic control.

### Renal outcomes in CANVAS

In the CANagliflozin cardioVascular Assessment Study (CANVAS) Program, a total of 10,142 participants with T2D and eGFR >30 mL/min/1.73m^2^ were randomly assigned to canagliflozin or placebo (58, 78). The CANVAS demonstrated significant cardiovascular safety and reported 14% lower rates of cardiovascular events among canagliflozin-treated participants compared to placebo-treated participants (59). Additionally, the composite of sustained doubled creatinine, ESRD or renal death occurred less frequently in the canagliflozin group than in the placebo group (hazard ratio: 0.53, 95% confidence interval [CI]: 0.33-0.84), along with consistent findings in prespecified patient subgroups. The annual eGFR decline was slower (slope difference 1.2 mL/min/1.73m^2^/year, 95% CI: 1.0-1.4), and the mean UACR was 18% lower (95% CI: 0.16-0.20) in participants treated with canagliflozin compared to those treated with placebo (**Table 4**). Furthermore, canagliflozin was associated with a reduced risk of sustained loss of kidney function, attenuated eGFR decline, and lower albuminuria, thus supporting a possible renoprotective effect in individuals with T2D (78).

At baseline, 2,039 (20.1%) subjects had an eGFR less than 60 mL/min/1.73m^2^, 71.6% of whom had a history of CVD. The effect of canagliflozin on the primary outcome, which was a composite of cardiovascular death, nonfatal myocardial infarction or nonfatal stroke, with other cardiovascular, renal and safety outcomes, was similar in people with CKD and those with preserved kidney function (*P* = 0.08). Most cardiovascular and renal outcomes were also similar across eGFR subgroups (eGFR < 60 and ≥ 60 mL/min/1.73m^2^) (58) (**Table 4**). The cardiovascular and renal outcomes with canagliflozin treatment were not affected by the baseline level of kidney function in T2D patients with a history of CVD at eGFR levels below 30 mL/min/1.73m^2^.

Although these results are consistent with those reported for empagliflozin, they provide additional support for mounting evidence that SGLT2 inhibitors (such as canagliflozin) can play an important role in delaying the development of diabetic nephropathy because the renal outcomes have been confirmed and assessed in this trial.

The Canagliflozin and Renal Events in Diabetes with Established Nephropathy Clinical Evaluation (CREDENCE) trial further assessed whether canagliflozin can slow renal failure in T2D patients with established kidney disease (eGFR of 30 to < 90 mL/min/1.73m^2^ and substantial albuminuria) (60, 79). The beneficial effect of canagliflozin on renal outcomes appeared to be consistent across the eGFR subgroups (30 to < 45, 45 to < 60 and 60 to < 90 ml/min/1.73m^2^) and baseline UACR (> 300 to ≤ 1000 mg/g, >1000 to < 3000 mg/g and ≥ 3000 mg/g) (60, 61) (**Table 4**). Subgroups with lower eGFR and higher baseline albuminuria had larger benefits for renal outcomes, although these patients were associated with the greatest risks for higher renal events (61, 79). Additionally, the effects of canagliflozin on renal outcomes were also observed even in patients with eGFR < 30 ml/min/1.73m^2^ (79). The CREDENCE study suggested that canagliflozin slowed the progression of diabetic kidney disease.

## Ipragliflozin

### Mechanistic studies of basic experiments

Ipragliflozin exerts renal protective effects in various animal models, including mice with either T1D (33) or T2D (33, 34, 80), progressive diabetic nephropathy (38) and spontaneously diabetic torii fatty rats (81). In both T1D and T2D mice, ipragliflozin exerted dose-dependent renoprotective effects *via* multifactorial pathways. Moreover, low-dose ipragliflozin reduced renal cortical hypoxia and the abnormal hemodynamics observed in early diabetic nephropathy. In addition, oxidative stress in tubular epithelia and glomerular podocytes was reduced with high-dose ipragliflozin (33). Additionally, in a model of diabetic nephropathy, ipragliflozin delayed the progression of diabetic nephropathy by attenuating glomerulosclerosis and interstitial fibrosis (38). In BTBR mice, ipragliflozin normalized diabetes-induced elevation in the renal pools of tricarboxylic acid cycle metabolites, as well as decreasing glomerular damage and tubulointerstitial fibrosis (34) (**Figure 1**, **Table 1**). It has also been shown that ipragliflozin administered intravenously can reduce kidney eGFR by altering local renal hemodynamics, which may be a possible mechanism underlying the renoprotective effect of ipragliflozin (81). In HFD-fed mice, the renoprotective mechanism of ipragliflozin appeared to be glucose-independent and may be attributed to improved mitochondrial abnormalities in renal tubules (80).

### Effects on renal markers in patients with T2D

A multicenter, open-label study evaluated the effects of ipragliflozin on diabetic nephropathy in patients with T2D (82, 83). After 24 weeks of ipragliflozin treatment, both the median UACR and mean eGFR were significantly decreased (*P* = 0.011 and *P* = 0.007, respectively) (82). During the prolonged post-trial follow-up period (104 weeks), eGFR was restored to near the baseline level at 104 weeks, although there was a transient decrease during the intervention period, and the medium UACR was significantly reduced compared to the baseline level (83). Thus, this study confirmed that ipragliflozin significantly reduced UAE in patients with diabetic kidney disease without decreasing eGFR.

Another study investigated the differences between two low-dose SGLT2is (canagliflozin and ipragliflozin) in patients with T2DM. Both demonstrated improved UAE (P < 0.05) and no changes in eGFR after the administration with SGLT2is for 24 weeks, which was different from previous studies (84).

### Renal outcomes in the LANTERN study

In the Long-Term ASP1941 Safety Evaluation in Patients with Type 2 Diabetes with Renal Impairment (LANTERN) study, patients with T2DM and mild to moderate renal impairment (RI) were assigned to 50 mg ipragliflozin or placebo once daily for 24 weeks and continued the treatment for a 28-week extended time period (total treatment duration: 52 weeks) (85). Ipragliflozin significantly improved HbA1c and fasting plasma glucose in patients with mild RI but did not improve hyperglycemia in T2D patients with moderate RI. Of note, eGFR showed a transient decline in the first two weeks, irrespective of RI severity, but was restored to baseline levels by 24 weeks in the ipragliflozin group. Unfortunately, no data were available regarding UAE in this study. In conclusion, these results suggested that ipragliflozin is an effective treatment option for T2DM patients with normal renal function or mild RI but not for those with moderate RI.

## Tofogliflozin

### Mechanistic studies of basic experiments

Tofogliflozin exerted renoprotective properties by suppressing renal injury. Treatment with tofogliflozin for 8 weeks significantly attenuated glomerular hypertrophy and decreased UACR in db/db mice compared with untreated db/db mice (86). It has been shown that tofogliflozin suppresses UAE and renal tubulointerstitial injury in KKAy/Ta diabetic mice (87). Additionally, tofogliflozin inhibited oxidative stress generation and had anti-inflammatory and anti-apoptotic effects in high glucose treated proximal tubular epithelial cells, suggesting beneficial effects of tofogliflozin on tubulointerstitial damage in diabetic nephropathy (21) (**Figure 1**, **Table 1**).

### Effects on renal markers in patients with T2D

A pooled analysis evaluated the effects of daily sodium intake (DSI) on eGFR in 775 Japanese T2D patients administrated with tofogliflozin (88). During 52 weeks of therapy, the mean eGFR significantly decreased at week 4 (-3.7 mL/min/1.73m^2^) and gradually increased from 4 to 52 weeks (+6.1 mL/min/1.73m^2^), thus demonstrating a considerable increase from baseline to week 52 (+2.4 mL/min/1.73m^2^). Additionally, a greater increase in eGFR at week 52 was significantly correlated with lower basal HbA1c and DSI levels. Thus, this open-label study suggested that the effect of tofogliflozin on eGFR may be closely associated with the handling of both glucose and sodium through SGLT2 in the renal tubule.

### Renal outcomes in the J-STEP/EL study

In the Japanese Study of tofogliflozin with T2DM Patients/Long Term (J-STEP/LT), 6,897 T2D patients were randomized to receive tofogliflozin (20 mg, once daily) (89, 90). After the 12 month observation period, both HbA1c and body weight significantly decreased from baseline levels by -0.76% and -2.73 kg, respectively (*P* < 0.0001). Except for the lowest eGFR subgroup, the HbA1c values of the other eGFR subgroups were significantly decreased (89). Moreover, a longer treatment with tofogliflozin showed that the HbA1c level was significantly reduced by -0.7% (*P* < 0.0001) and body weight decreased by -2.95 kg from baseline to week 104 (*P* < 0.0001) (90). Unfortunately, no data were available regarding renal outcomes in this analysis.

## Luseogliflozin

### Mechanistic studies of basic experiments

Luseogliflozin showed renoprotective effects in various animal models, including a non-diabetic model of renal ischemia/reperfusion injury (39) and a type 2 diabetic nephropathy (T2DN) rat model (40, 45, 91). Moreover, luseogliflozin prevented renal interstitial fibrosis by increasing vascular endothelial growth factor (VEGF)-A expression after ischemia/reperfusion injury in non-diabetic mice (39). In animal models of T2DN, luseogliflozin prevented the decrease in GFR and greatly reduced the degree of glomerular injury, renal fibrosis and tubular necrosis partially by inhibiting HIF-1α accumulation (40, 45) (**Figure 1**, **Table 1**). In diabetic db/db mice, the renoprotective effects of luseogliflozin may be independent of glucose-lowering effects but associated with the altered glomerular distribution and size by affecting oxygen metabolism and humoral factors (91).

### Effects on renal markers in patients with T2D

In a retrospective study, 238 patients with T2D were divided into 3 groups based on the eGFR levels and administrated with 2.5 mg luseogliflozin once daily (92). In all of the subjects, changes in eGFR from baseline exhibited a negative correlation with the basal eGFR. Specifically, the eGFR was significantly decreased from baseline levels in both the high and normal eGFR groups, whereas no significant difference was observed in eGFR with the low eGFR group. In addition, the UACR change was larger in the low eGFR group than in the high and normal group, although there was no statistical significance (92). As an acute decrease in eGFR is a common effect of SGLT2is, another study investigated the relationship between acute decreases in eGFR and the course of eGFR after initiating luseogliflozin treatment (93). In this analysis of four 52-week trials, T2D patients were given luseogliflozin (2.5 mg and 5 mg daily) and acute changes in eGFR during the first 2 weeks were recorded. A greater acute decline in eGFR was observed in those with higher baseline eGFR and the course of eGFR thereafter was maintained regardless of the degree of the acute changes (93).

## Discussion

At present, the choice of anti-diabetic medications for clinicians is not limited to glucose-lowering efficacy, but needs to consider other aspects, such as the risk of hypoglycemia, the impact on body weight and blood pressure, the effects on the heart and kidney, safety and cost (10, 11, 94). The impact of these aspects with the treatment of anti-diabetic drugs will become increasingly important in clinical practice, especially in preventing macrovascular (cardiovascular disease) and microvascular (diabetic nephropathy and diabetic retinopathy) complications associated with T2D (11). To date, there have been many large-scale clinical trials on the renal effect of glucose-lowering drugs, which has become a highly-researched topic. Drugs with both hypoglycemic and renal protective effects must have more advantages in clinical use.

The effects of sulphonylureas on renal outcomes have rarely been studied, which mainly under real-life conditions (95). In the milepost United Kingdom Prospective Diabetes Study (UKPDS), intensive glucose control with insulin or sulphonylureas reduced the relative risk of microalbuminuria or proteinuria by 33% and the proportion of patients doubled their plasma creatinine levels at 12 years (0.91% vs. 3.52%, respectively; *P* = 0.0028) (96, 97). The renal outcomes with sulphonylureas were poorer than those metformin in some observational retrospective studies (98, 99). Hence, the effects of sulphonylureas on renal outcomes in patients with T2D remains unclear. Metformin is the initial pharmacological option for the treatment of type 2 diabetes. In recent years, metformin has been shown to have protective effects on diabetic kidneys through anti-inflammatory and anti-oxidative stress effects, which are independent of its glucose-lowering effect (100–103). However, more clinical trials are needed to further confirm these research findings. Clinical trials of GLP-1RA and DPP-4is have also provided some informative results, especially the LEADER trial for liraglutide (104, 105), the SAVOR-TIMI 53 trial for saxagliptin (106, 107) and the TECOS trial for sitagliptin (108). The LEADER study (104) showed that liraglutide 1.8 mg once daily can reduce urinary protein, slow down the decline rate of eGFR and significantly reduce the risk of microvascular events compared with the placebo group, thus suggesting that GLP-1RA has renal protective effects. DPP-4is did not identify such positive renal function outcomes, when considering saxagliptin in the SAVOR-TIMI 53 trial (106, 107) or sitagliptin in the TECOS trial (108). This may be due to the increase in GLP-1 levels by incretin analogs (such as liraglutide) rather than due to incretin enhancers (such as DPP-4is), and the positive effects of GLP-1 on endothelial dysfunction, inflammation and oxidative stress exceed its positive effects on body weight and blood pressure (109, 110). However, it should be noted that in both SAVOR-TIMI 53 and TECOS trials, as in other placebo-controlled, non-inferiority safety trials, balance blood glucose control is the goal (although it is not fully achieved). Therefore, more RCT studies should be performed to verify the effects of DPP-4 inhibitors on the kidney.

Recent trials with the effects of SGLT2 on the kidney seem to be even more impressive, especially the EMPA-REG OUTCOME trial for empagliflozin, the CANVAS trial for canagliflozin and the DECLARE-TIMI 58 trial for dapagliflozin. These studies showed a significant reduction in a composite of renal outcomes (the doubling of serum creatinine, progression to macroalbuminuria, the initiation of renal replacement therapy and death from renal disease) in T2D patients with CVD compared with in the placebo group. As has been previously mentioned, these effects can be explained neither by a mild improvement in blood glucose control (a scheme aimed at achieving blood glucose equipoise) (111) nor by a slight decrease in arterial blood pressure (112). In addition to exerting renal protection through anti-inflammatory, anti-oxidant stress and anti-fibrosis effects, the specific intrarenal effects produced by tubuloglomerular feedback seem to be the most likely explanation for the better renal outcomes of SGLT2 thus far (113, 114). The specific mechanism is not completely clear and requires further research.

The favorable renal effects of large-scale clinical studies with SGLT2is have been published or are currently underway, with the goal of identifying a new treatment strategy for T2DM. The preliminary results of several clinical trials have shown a significant reduction in proteinuria and eGFR, the need for renal replacement therapy or death due to renal disease. Experimental data from animal models have shown that SGLT2is exert renal protection through various mechanisms other than glucose control. Although more studies are needed, the current results are likely to provide a foundation for the application of SGLT2is in patients with diabetic nephropathy, as well as for the prevention of renal dysfunction, and for the management of renal disease in patients without diabetes.

## Conclusions

As a new class of antihyperglycaemic medications, SGLT2i is increasingly being used in the treatment of T2D. Preliminary findings from multiple clinical studies and large-scale clinical studies with cardiovascular endpoints devoted to SGLT2is have consistently shown significant decrease in albuminuria and stable eGFR that was independent of changes in glucose control. In animal studies, SGLT2i can protect kidney by suppressing RAS, decreasing intraglomerular pressure, improving mitochondrial dysfunction, alleviating oxidative stress, improving fibrosis, and inhibiting apoptosis, and eventually it is proposed to have a potential impact on DN. According to the fingdings, SGLT2is can be considered as a promising agent for the prevention, attenuation or even reversal of DN. However, studies specifically for renal endpoints with SGLT2is are still lacking and more clinical trials and pre-clinical studies are required.

## Author contributions

XS collected data, analyzed data, and wrote the manuscript. GW reviewed and edited the manuscript. All authors contributed to the article and approved the submitted version.

## Conflict of interest

The authors declare that the research was conducted in the absence of any commercial or financial relationships that could be construed as a potential conflict of interest.

## Publisher’s note

All claims expressed in this article are solely those of the authors and do not necessarily represent those of their affiliated organizations, or those of the publisher, the editors and the reviewers. Any product that may be evaluated in this article, or claim that may be made by its manufacturer, is not guaranteed or endorsed by the publisher.
